# Three-Dimensional Analysis of Round Window Membrane in the Chinchilla Model with Acute Otitis Media Induced with *Streptococcus Pneumoniae* 7F

**DOI:** 10.4274/tao.2021.5998

**Published:** 2021-03-26

**Authors:** Nevra Keskin Yılmaz, Hasan Albasan, Mehmet Kazım Börkü, Michael Mauro Paparella, Sebahattin Cüreoğlu

**Affiliations:** 1 Department of Internal Medicine, Ankara University, Faculty of Veterinary Medicine, Ankara, Turkey; 2 Department of Otorhinolaryngology, Head and Neck Surgery, University of Minnesota Medical School, Minneapolis/Minnesota, USA; 3 Pet Depot Veterinary Group, La Verne, California, USA

**Keywords:** Acute otitis media, pathology, cochlear round window, Streptococcus pneumoniae, cellular morphology

## Abstract

**Objective:**

The purpose of this study was to investigate the morphological changes of round window membrane (RWM) in chinchillas with *Streptococcus pneumoniae (S. pneumoniae)* serotype 7F induced acute otitis media (AOM) by two dimensional (2D) and three dimensional (3D) measurements.

**Methods:**

Temporal bone specimens taken from 12 chinchillas were divided into two groups. The control group consisted of healthy animals that were injected with intrabullar saline. The subjects in the experimental group were induced with AOM by intrabullar injection of *S. pneumoniae* 7F. The 2D and 3D measurements of RWM were compared between the groups.

**Results:**

Dramatic changes were noted in the RWM of the experimental group compared to the control group. The thickness [mean ± standard deviation (SD)] of the RWM was significantly (p<0.05) increased in the experimental group compared to the control group by 2D measurements taken at three different points of RWM. Moreover, 3D measurements revealed that the volume (mean ± SD) of RWM was significantly (p=0.009) increased in the experimental group.

**Conclusion:**

The results of our study, which indicated significant change in RWM in both 2D and 3D measurements, may shed light on the relationship between AOM and inner ear diseases. Based on our results, we recommend evaluating 3D analyses of RWM, which provide useful data, to better understand the changes in the membrane.

## Introduction

Acute Otitis media (AOM), which defines the inflammation of the tympanic cavity, the Eustachian tube, the mastoid process and the mucoperiosteum surrounding the mastoid air cells, is recognized as one of the most important animal and human health threatening infectious diseases across the world (1, 2). Given the anatomical localization of the ear, AOM should be evaluated in terms of many different complications, extracranial and/or intracranial (3, 4, 5, 6).

The round window membrane (RWM), which separates the middle ear and the scala tympani of the basal turn of the cochlea, has an important role in several pathologies involving the middle and the inner ears in that it is a semi-permeable membrane allowing bacterial toxins, inflammatory mediators, and antibiotics to pass through (7, 8). As RWM takes part in the body’s defense system and plays a role in the transport of mechanical energy between the ear ossicles and the inner ear fluid; the structural changes that occur in the RWM with the thickening of the membrane give rise to the pathology of many diseases involving the middle and the inner ears including hearing loss (3, 8, 9, 10). Therefore, analyzing any changes in RWM plays an important role in determining the pathogenesis of possible complications that may arise from otitis media and labyrinthitis.

Three-dimensional (3D) analysis of different anatomical regions is a field that is increasingly investigated as it helps to better understand and evaluate the detailed structure of the focused area. Although many studies have been conducted to examine the changes in the RWM, to our knowledge, there is no study that examined the morphological changes in the membrane in three dimensions and revealed the relationship of these measurements with the two-dimensional (2D) analyses of the entire surface of the membrane. The aim of this study was to assess any morphological changes of the RWM by 2D and 3D measurements in the chinchilla model with *Streptococcus pneumoniae* (*S. pneumoniae*) serotype 7F induced acute otitis media (AOM).

## Methods

### Design and Setting

The study used 12 Chinchillas divided into two groups. In the experimental group (n=6), the superior bullas of the chinchillas were bilaterally shaved after anesthesia with ketamine-HCl (20 mg/kg, intramuscular). After the region was cleaned in accordance with the asepsis-antisepsis rules, *S. pneumoniae* serotype 7F (2x10^1^ bacteria/bulla) was injected bilaterally into the middle ears of the chinchillas. The bacteria used to create AOM was obtained by preparing a sample from a patient diagnosed with OM originating from *S. pneumoniae* serotype 7F at the University of Minnesota Medical School, Department of Otorhinolaryngology Head and Neck Surgery, by culturing and reproducing it in a blood agar. Otoscopic examination was performed in all subjects during the study and the animals were euthanized with the injection of high-dose ketamine-HCl seven days after inoculation. To our knowledge, this is the first study to make the 3D analysis of the chinchilla RWM. Therefore, right and left temporal bone samples were compared to each other to reveal whether there was difference between the contralateral ears in the experimental group; and the right temporal bone samples of the two groups were compared to determine the difference between the experimental and control groups. To ensure uniformity in the comparisons between the two groups, the same amount of saline solution was also injected into the right ears of the chinchillas in the control group (n=6) and the subjects were euthanized seven days after the injection.

After the animals were euthanized, all temporal bones in both groups were removed for further processing and histopathological examination. This temporal bone study was approved by the Institutional Review Board of the University of Minnesota (#0206M26181).

### Histopathological Assessment

After euthanasia, all temporal bones were removed, perfused in Heidenhain’s Susa solution, fixed in formalin, decalcified, and embedded in celloidin, and serially sectioned in the horizontal plane at a thickness of 20 µm. Every 10^th^ section was stained with hematoxylin-eosin and studied by light-microscopy using digital camera and image analysis software (SPOT Advanced; SPOT Imaging Solutions, Sterling Heights, MI, USA).

The round window thickness was determined by taking the average of the measurements made from three to five sections where the membrane was the widest. Each section was measured from three different points. The first measurement was made from the center of the round window, while the other two measurements were performed 0.2 mm anterior and inferior (10, 11).

We performed 3D reconstruction of the RWM by using the reconstruction software (Amira 3D Software for Life Sciences; FEI, Hillsboro, OR, USA) on the scanned sections with a high-resolution scanner (PathScan Enabler IV; Meyer Instruments, Houston, TX, USA) including the mid-modiolar level, as well as on the adjacent two to four sections where the RWM is best observed and could be digitized.

### Statistical Analysis

To determine the sample size, we conducted power analysis with G*Power software version 3.1.9.2. (University of Kiel; Kiel, Germany) by taking the type I-α error rate 0.05 and calculating the power of the test as 0.80 and the groups were evaluated for statistical significance by using the Mann-Whitney U test with MedCalc Statistical Software version 12.7.7 (MedCalc Software Bvba; Ostend, Belgium). Significance was defined as p<0.05.

## Results

**Figure 1:**
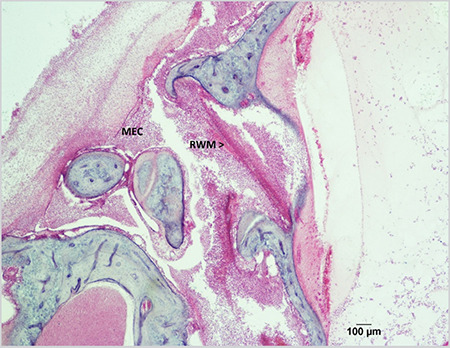
Round window niche is filled with inflammatory cells accompanied with thickened round window membrane (x2) MEC: Middle ear cavity, RWM: Round window membrane (H&E), H&E: Hematoxylin-eosin

The results of our study revealed severe inflammatory cell infiltration, bacterial invasion, and dense fibrous structure in the middle ear cavity in the experimental group. Moreover, the round window niche was filled with serous exudate and polymorphonuclear leukocytes (PMNs) were seen in intense amounts in all temporal bone specimens in the experimental group. Severe inflammatory cell invasion was also observed in the scala tympani of the lower basal turn of the cochlea (Figure 1).

**Figure 2a:**
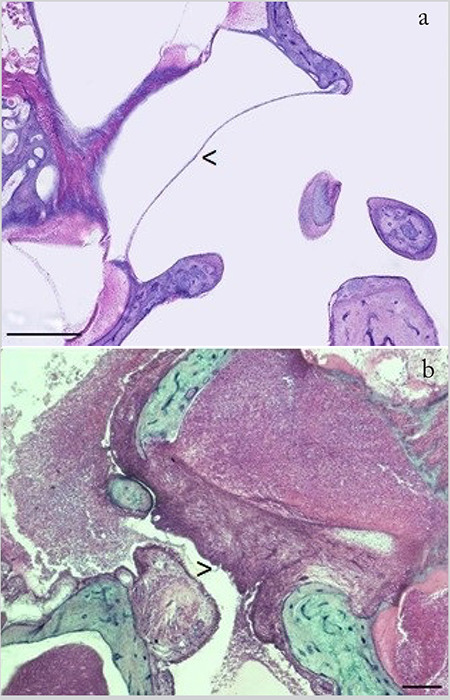
The normal structure of the RWM in the control group, b. Severe thickening of RWM in the experimental group due to intracellular edema and cell proliferation. Arrow heads: RWM (scale bar: 500 μm, H&E) RWM: Round window membrane, H&E: Hematoxylin-eosin

When examined morphologically, there were no differences between the right and the left RWMs in the experimental group, whereas there were significant changes in the experimental group compared to those in the control group (Figure 2a). RWM was found to be thicker at the anterior and posterior ends of the RWM and thinner at the center in the control group and the membrane was positioned convexly towards the scala tympani of the basal fold of the cochlea. In the experimental group, however, there was pronounced thickening of the membrane both in the right and the left ears that was most intense on the epithelial surface and edematous enlargement in the fibrous middle layer (Figure 2b). The convex shape of the membrane was more flattened in the experimental group.

**Figure 3:**
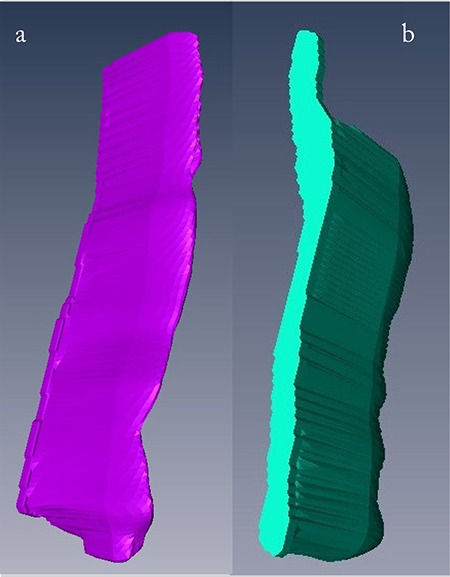
Representative photos of 3D reconstruction of the RWMs. a. Healthy RWM from control group, b. Thickened RWM from experimental group RWM: Round window membrane

The membrane thickness measurements taken at three different points did not reveal significant differences between the right and the left ear measurements in the experimental group in 2D analysis (p>0.05). However, we found significant differences between the experimental and the control groups. In the measurements, anterior was 17.6 µm, center was 15.32 µm, and inferior was 18.31 µm in the control group, while anterior was 31.26 µm, center was 32.52 µm, and inferior was 36.57 µm in the experimental group. Membrane thicknesses were significantly higher in the anterior (p=0.009); in the center (p=0.015), and in the inferior (p=0.041) of the experimental group compared to those of the control group. Moreover, the mean thickness of the three points [mean ± standard deviation (SD)]; 33.5±16.7 µm) in the experimental group was significantly (p=0.009) higher compared to those (mean ± SD; 16.4±2.4 µm) of the control group. In the 3D analysis, the mean volume of the RWM was significantly (p=0.009) higher (mean ± SD; 0.106±0.038 mm³) in the experimental group compared to that (mean ± SD; 0.066±0.008 mm³) of the control group (Figure 3). As in the 2D analysis, we found no significant differences in 3D analysis between the right and the left ears in the experimental group (p>0.05).

## Discussion

AOM is a commonly seen disease which decreases the quality of life, requires patients to frequently present to clinics because of high recurrence rates and complications. The round window niche is a region that has been studied by many researchers due to its proximity to vital structures such as the tympanic segment of the facial nerve, the jugular vein, and the RWM that forms the middle ear-perilymph barrier (12, 13, 14).

In this study, we observed that after inoculating the bacteria via intrabullar injection into the middle ear, dense accumulation of purulent exudate occurred, especially in front of the round window niche and in the scala tympani of the basal turn of the cochlea. Previous studies have shown that free radicals, bacterial exotoxins, antioxidants, antibiotics, local antiseptics, and bacteria in the round window niche can penetrate the RWM and pass through the inner ear, and the material from the middle ear cavity may spread to inner ear. If this material affects the inner ear, it may lead to the development of sensorineural hearing loss, labyrinthitis and even otogenic meningitis(3, 8, 12, 15, 16, 17, 18).

In our study, the cross-sections of RWM showed differences in the shape of the membrane in the experimental group. While a normal chinchilla RWM has a more convex shape, the RWM of the infected ears showed a more flattened shape in our study, in accordance with the findings of previous studies (10, 12). Most researchers assume that this difference in the shape of RWM is related with the negative middle ear pressure due to inflammatory cell and exudate infiltration in the tympanic cavity due to acute onset of OM as well as with the inflammatory changes in the layers of the membrane (10, 19). But in all circumstances, the contraption involving the change in the shape of RWM may affect the whole interaction between the middle and the inner ears, hence the hearing mechanism.

Although the pathologies caused by *S. pneumoniae* in the RWM have been examined in many histopathology studies, to our knowledge, the quantitative data on membrane thickness have not been studied (12, 20). Considering that the number and species of the microorganisms used, and the duration of inoculation significantly affect the changes in membrane thickness, a better understanding of the pathological process by quantitative measurements support the results of the experiments*. *In the presented study, we found in the 2D measurements that the thickness of the RWM was significantly higher in the experimental group compared to those in the control group. In accordance with our results, Jiang et al. (10) observed that the membrane was significantly thickened in the chinchillas with AOM created by *Haemophilus influenzae *inoculation compared to the control group in four days after inoculation. In both their and our studies, the thickening of the RWM, which may be explained by the intracellular edema and cell proliferation occurring in the membrane, may be interpreted as part of the body’s defense system to protect the inner ear and other related structures from inflammation in the middle ear. However, the exact mechanism to understand the underlying pathology should be evaluated further.

The thickening of the RWM is directly related with the changing mechanism of membrane permeability. Numerous studies show that the permeability of RWM relatively increases in the early stages of OM, whereas it tends to decrease in the later stages (12, 20, 21, 22, 23). In a study conducted on 25 cats with OM with Eustachian tube obstruction, it was observed that the transition within three days following the obstruction was similar to the transition from normal RWM, but the permeability of the membrane was greatly reduced after one to two weeks of occlusion (21). Similarly, a study in guinea pigs inoculated with *Pseudomonas aeruginosa* showed a gradual increase in permeability one week after OM was induced; however, permeability decreases when the inflammatory processes associated with OM are prolonged for more than two weeks (22). Based on these studies and our findings, it could be concluded that the increase in RWM permeability in the early period of OM may be due to the degenerative changes in the epithelial cells of the membrane. As the inflammatory reaction is prolonged, however, permeability decreases due to increased fibrosis in the middle layer of the membrane and effusion in the round window niche on either side of the membrane and the presence of PMNs (21, 22).

3D reconstruction of the temporal bone is an increasingly used method as it helps the surgeon to better understand the anatomical structure in the diagnosis and treatment of otologic diseases and in ear operations (24, 25). Although it is possible to distinguish and analyze the anterior, inferior, and center of the RWM in 2D analyses on temporal bone sections, in 3D analysis the membrane cannot be examined in this way and can be analyzed as a single structure. In the present study, however, when the temporal bone samples in the experimental group were examined, the thickening that occurred at all three points of the membrane could be clearly observed in 3D visuals. Therefore, performing a 3D reconstruction, enabled us to display the status and understand the changing angle of the RWM, and helped to track the thickness of the membrane.

Understanding the anatomical position of the RWM and the changes in its structure in three dimensions has importance for pharmacokinetic research (26). Recent studies show that local drug delivery is a more efficient option than systemic antibiotic therapy in the treatment of inner ear diseases (26, 27). In this direction, as it acts as a gateway between the middle and the inner ears, and because it is a metabolically active membrane, RWM may play an active role in the transfer of drugs intended to reach the inner ear (26, 27, 28). However, changes in the permeability of the membrane can complicate the manipulation of the drugs that are planned to be delivered to the inner ear, and the efficiency of the treatment may decrease (28). Therefore, determining the morphological changes and the 3D display of the angle and the location of the membrane may affect the treatment methods and results. Although there are only a few reports studying the reconstruction of RWM, 3D analysis of the membrane by using in-vivo visualization techniques may be a helpful method to better understand and analyze the morphology of the membrane and related structures and to direct the treatment protocols.

## Conclusion

In our study, it was determined that the results obtained by the 3D measurements of the RWM were compatible with those of 2D measurements. In parallel with 2D measurements, 3D reconstruction results also showed that the volume of RWM in the experimental group was increased compared to those in the control group. These results show that the measurement of the membrane with 3D methods for pathologic illumination may significantly contribute to the outcomes in patients whose RWM permeability or stiffness are thought to be affected.

**Main Points**

• This study provides a detailed histopathological description and quantitative information on the morphological changes of the RWM in AOM induced with *S. pneumoniae* serotype 7F.

• The results showed that the shape and the thickness of RWM changes within seven days after *S. pneumoniae* injection into the middle ear.

• The results of 2D and 3D analyses of the RWM were compatible; and the 3D reconstruction of the RWM may be a helpful tool to understand and analyze the changes in the membrane caused by AOM.
